# Electrically programmable magnetoresistance in $\text{AlO}_{x}$-based magnetic tunnel junctions

**DOI:** 10.1038/s41598-021-84749-x

**Published:** 2021-03-16

**Authors:** Jhen-Yong Hong, Chen-Feng Hung, Kui-Hon Ou Yang, Kuan-Chia Chiu, Dah-Chin Ling, Wen-Chung Chiang, Minn-Tsong Lin

**Affiliations:** 1 Department of Physics, Tamkang University, New Taipei City, 25137 Taiwan; 2 Department of Physics, National Taiwan University, Taipei, 10617 Taiwan; 3 Department of Optoelectric Physics, Chinese Culture University, Taipei, 11114 Taiwan; 4 Institute of Atomic and Molecular Sciences, Academia Sinica, Taipei, 10617 Taiwan

**Keywords:** Spintronics, Magnetic properties and materials

## Abstract

We report spin-dependent transport properties and I–V hysteresis characteristics in an $\text{AlO}_{x}$-based magnetic tunnel junction (MTJ). The bipolar resistive switching and the magnetoresistances measured at high resistance state (HRS) and low resistance state (LRS) yield four distinctive resistive states in a single device. The temperature dependence of resistance at LRS suggests that the resistive switching is not triggered by the metal filaments within the $\text{AlO}_{x}$ layer. The role played by oxygen vacancies in $\text{AlO}_{x}$ is the key to determine the resistive state. Our study reveals the possibility of controlling the multiple resistive states in a single $\text{AlO}_{x}$-based MTJ by the interplay of both electric and magnetic fields, thus providing potential applications for future multi-bit memory devices.

## Introduction

Manipulation and control of transport properties of spin-polarized electrons in nano-structured magnetic multilayers, such as Giant Magnetoresistance (GMR) in spin valves and Tunneling Magnetoresistance (TMR) in magnetic tunnel junctions (MTJs)^1–4^, offer versatile possibilities for developing novel spintronic applications in information storage and memory devices. However, to meet the increasing demand for faster, smaller and lower energy consumption in the relevant technologies, the development of devices equipped with multi-functionalities and nonvolatile electronic properties is of intensive interest. Recently, there have been a lot of efforts aiming at the electric-field control of magnetism in MTJs to manipulate their magneto-electronic transport properties^5–7^. Such a strategy could lead to desirable ultra-low energy consumption, a demand increasingly sought after in nano-scaled devices. Furthermore, by introducing a capacitive charge accumulation at the multilayer interfaces, the density of states at the Fermi level could be altered by an external electric field, which in turn could lead to the changes of magnetism and magnetic anisotropy^8–10^.

In contrast, another type of electric-field controlled, nonvolatile resistive switching (RS) effect based on the reversible resistive changes between the high and the low resistance states (HRS and LRS) of a metal/insulator/metal structure (identical to an MTJ, except the use of a non-ferromagnetic metal as the electrodes) is regarded one of the most promising new memories with the advantages of high integration, low power consumption, high read-write speed, non-volatility and compatibility with the existing CMOS (Complementary Metal-Oxide-Semiconductor) technology^11–13^. It would be an important step towards multi-functionality if both resistive switching and magnetoresistance can be integrated into a single device and its multiple states can be electrically/magnetically controlled at the same time. Previous studies have investigated the RS effect in $\text{AlO}_{x}$ and MgO-based MTJs^14–16^; nevertheless, after the electroforming of the resistive memory operation process, it is difficult to simultaneously obtain both observable on-off and MR ratios. In most cases either the TMR is absent or the on-off ratio is suppressed^17,18^. In this study, we demonstrate that both resistive switching and magnetoresistance in an $\text{AlO}_{x}$-based MTJ can be achieved via electric/magnetic control of the resistance states. The bipolar resistive switching ratio between HRS and LRS is up to 202${\%}$ and the device shows a simultaneous tunneling MR ratio of 10${\%}$ and 4${\%}$ at HRS and LRS, respectively, promising a multi-bit memory of four distinctive memory states.

## Experimental section

**Figure 1:**
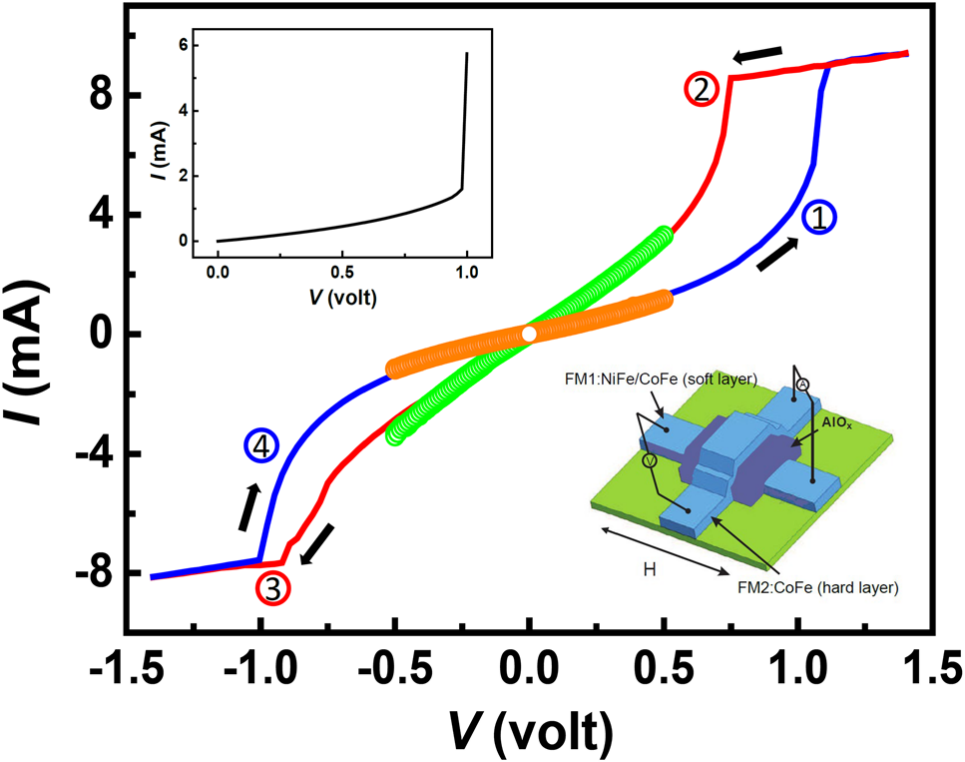
Current–voltage (I–V) curves of a NiFeCoFe/$\text{AlO}_{x}$/CoFe MTJ structure with arrows indicating the directions of the voltage sweep. The different colors of the I–V curves represent different directions and ranges of the sweep (see details in the text). The upper-left inset shows the electroforming process, in which a relatively high positive bias is applied on the CoFe electrode. The lower-right inset shows the schematic of the device structure.

The structure of the $\text{AlO}_{x}$-based MTJ is stacked in the sequence of NiFe (15 nm)/CoFe (10 nm)/$\text{AlO}_{x}$ (1.5 nm)/CoFe (35 nm) and patterned in a crossed-bar configuration, with the bottom NiFe/CoFe layer serves as the soft ferromagnetic (FM) electrode and the top CoFe as the hard FM electrode, as illustrated in the lower-right inset of Fig. 1. The entire fabrication process was executed in a UHV sputtering chamber with a base pressure of $1\times 10^{-8}$ mbar. The $\text{AlO}_{x}$ layer was prepared by nature oxidation of Al followed by an $\text{O}_{2}$ plasma treatment at 3 mbar for 20 seconds. Details of the sputtering and the patterning processes can be retrieved in our previous work^19^. The current–voltage (I–V) characteristics were measured at various voltages and the magnetoresistance was measured in the Current-Perpendicular-to-the-Plane (CPP) configuration using the four-point-probe method with the bias voltage applied on the top electrode while the bottom electrode was grounded, whereas the temperature dependence of the resistance was measured in a Quantum Design Physical Property Measurement System (PPMS).

## Results and discussion

**Figure 2:**
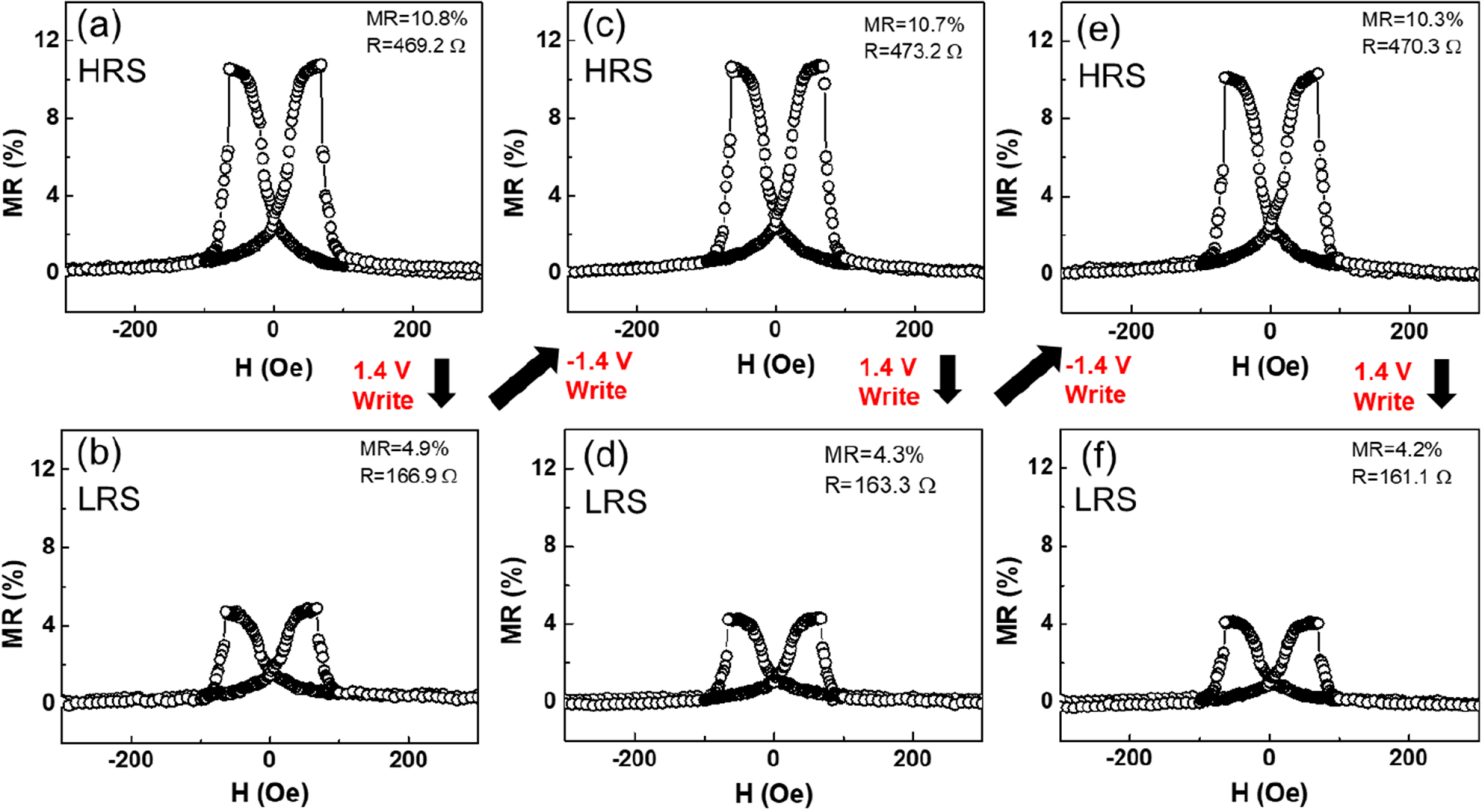
Sequential measurements of magnetoresistance of the reported MTJ sample. Pre-applied bias voltages and the sequence for setting the resistance states are indicated by red letters and black arrows in between the graphs whereas the MR measurements were carried out at 5 mV. The bias of 1.4 V (− 1.4 V) sets the device to LRS (HRS) where lower (higher) MR is obtained.

The current–voltage (I–V) curves of the device after electroforming are shown in Fig. 1, which exhibit reversible, nonvolatile bipolar resistive switching characteristic. The room temperature MR effect can be observed at both HRS and LRS (see the discussion of Fig. 2 below). The newly fabricated device shows a high resistance ($\sim 1.3\text{ k}\Omega$) in its pristine state. After the electroforming process at $\sim 1$ volt (see the upper-left inset of Fig.1), the device switches from the pristine state to the low resistance state (LRS). Here a compliance current (ICC) of 9.4 mA is applied to avoid excessive current flow and to prevent the irreversible hard breakdown. The sudden increase of current level during the electroforming process causes localized heating and the formation of oxygen vacancies in the $\text{AlO}_{x}$ barrier, producing a new remnant insulating gap between the two ferromagnet electrodes along with an irreversible decrease of resistance from the pristine state^20^. Following the electroforming process, the voltage is swept between + 1.4 and − 1.4 V in a cyclic manner with the arrows in Fig. 1 indicating the directions of the voltage sweeps. When increasing the voltage from 0 to + 1.4 V (step 1 in Fig. 1) during the sweep, the current increases abruptly at $\sim 1.1\text{ V}$ which transits the device to low resistance state (LRS). From 1.1 to 1.4 V, despite an applied ICC of 9.4 mA, a flattened slope is present which can be attributed to the self-rectifying characteristic of $\text{AlO}_{x}$^21^. On the negative bias side, the same behavior is observed at $\sim -1.0\text{ V}$ which switches the device from LRS to HRS (step 3 in Fig. 1). Here we designate LRS as the “ON” or “‘1” state, and HRS as the “OFF” or “0” state. The on-off ratio of the device is 202$\%$ at 0.9 V. To further demonstrate the non-volatility of the resistive switching, a test was performed in such a way that the applied voltage was suddenly turned off at 1.4 V. An I–V curve was taken immediately after between + 0.5 and − 0.5 V (represented by the green open circles in the figure). As shown in Fig. 1, the curve follows closely the footprint of the major loop. The same procedure applied on the negative side results in a similar behavior (see the orange open circles), showing that the resistance states are reproducible. The I–V and the corresponding MR measurements have been performed for more than 100 cycles, during which the MR variation is within 10$\%$ and 29$\%$ for HRS and LRS, respectively. The overall decrease of MR after 100 cycles is about 3$\%$ for HRS and 11$\%$ for LRS. These systematic results suggest a robust nonvolatile memory characteristic of the device.

Figure 2 shows the magneto-transport characterization of the $\text{AlO}_{x}$-based MTJ, in which the cycling of junction resistance is plotted against the applied magnetic field. The resistance curves display the typical pseudo spin-valve type characteristics at room temperature (RT) with a magneto-resistive (MR) ratio defined as

1 $$\begin{aligned} \frac{\Delta R}{R_{P}}=\frac{R_{AP}-R_{P}}{R_{P}} \end{aligned}$$

where $\text{R}_{P}$ and $\text{R}_{AP}$ depict the resistances when the magnetizations of the FM electrodes are in the parallel and the antiparallel configurations, respectively. Electrical control of magnetoresistance has been regarded an alternative way to extend the functionality of a memory device^22^. To better understand the interplay between the magneto- and the resistive switching effects in the $\text{AlO}_{x}$-based MTJ, the device was set from one resistance state to another by applying a voltage at − 1.4 V (HRS) and + 1.4 V (LRS), and the magnetoresistances were measured subsequently at 5 mV. Figure 2a shows the MR loop of the MTJ at HRS, with a resistance of 469 $\Omega$ and an MR ratio of 10.8$\%$. Upon applying the positive 1.4 V bias voltage, the resistance decreases from 469 $\Omega$ to 167 $\Omega$ (LRS). The subsequent MR measurement at 5 mV shows a reduced MR ratio of 4.9$\%$ (see Fig. 2b). Repeating the procedure indicates that the effect is reversible. Figure 2c gives a resistance of 473 $\Omega$ (HRS) and an MR ratio of 10.7$\%$ upon setting the bias voltage back to -1.4 V. Figure 2d–f show that by further switching the bias between − 1.4 and + 1.4 V, the dual resistance states and MR states are both preserved. The same behavior was observed in other MTJs made in the same run under identical conditions. In a set of 19 simultaneously-made samples, 13 samples exhibit a uniform distribution of junction resistances with variation within 10$\%$ in the pristine state, while the other 6 samples show larger discrepancies due to the edge effect of the deposition rate. By carefully controlling the compliance current during the electroforming process, the resistance of either HRS or LRS can be controlled within 20$\%$ from sample to sample. This variation is likely due to the differences of oxygen vacancies in $\text{AlO}_{x}$. Figure 2 presents strong evidences for multifunctional operation in these MTJ devices. Both junction resistance and magnetoresistance can be manipulated reversibly by the interplay of electric/magnetic fields in a nonvolatile manner. The results lead directly to the possibility of integrating multiple-state memories into a single device, which goes beyond the binary (0–1) limit.

**Figure 3:**
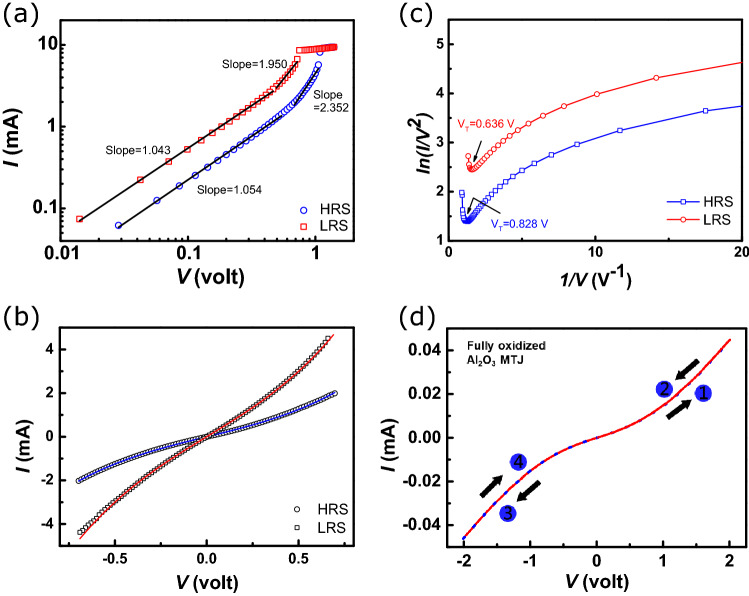
(**a**) The linear fits (black solid lines) of the I–V curves in double-logarithmic scales for the positive voltage region. (**b**) The fitting results at low bias voltage using the Simmons model. The plot in (**c**) indicates the transition from direct tunneling (DT) to Fowler-Nordheim (FN) tunneling at $\text{V} = \text{V}_{T}$ (see text). (**d**) The I–V curve of a fully oxidized $\text{Al}_{2}\text{O}_{3}$ MTJ, which shows no resistive switching.

To further investigate the mechanism of resistive switching in our device, we re-plot the positive sweep region of Fig. 1 in double-logarithmic scales (see Fig. 3a), in which the fitting results are indicated by black lines. In the low-voltage regime (0–0.5 V) of HRS, the curve exhibits a linear relation between current and voltage with a slope of $\approx$ 1.054. When V $\ge$ 0.66 V, the curve becomes nonlinear, and a linear regression results in an enhanced slope of $\approx$ 2.352. By further increasing the voltage, the current saturates to a constant value. Upon passing the threshold voltage ($\sim$1.1 V), the device switches from HRS to LRS. When reversing and decreasing the voltage, the I–V curve goes through a nonlinear region (above 0.48 V, with a regression slop of 1.950) and a linear region (from 0.48 to 0 V, with a slope of 1.043). The power law fittings ($\text{I }\propto \text{ V}^{m}$) to both the HRS and the LRS curves yield two distinctive regimes, i.e. the low bias-voltage regime with m $\approx$1 which exhibits Ohmic characteristics (I $\propto$ V), and the high bias regime with $\text{m}\approx 2$ which obeys the Child’s law ($\text{I }\propto \text{ V}^{2}$)^23^. The conduction mechanism in the low bias regime is dominated by the thermally generated free electrons, such as the Schottky or the thermionic emission, which is one of the most often observed conduction mechanisms in oxides and semiconductors^24^. After which, the field exceeds the square-law onset voltage and the electron density gradually surpasses the equilibrium concentration and dominates the conduction in the higher bias regime. Such a behavior is common in oxides consisting of a discrete trapping level with both trap-unfilled and trap-filled transport characteristics^23^.

Figure 3b shows the fitting results (blue curve for HRS and red curve for LRS) of the low-voltage I–V curves using the Simmons equation $I= aV + bV^{3}$, where *a* and *b* are two barrier shape-related coefficients^25^. Assuming a rectangular type barrier, both the HRS and the LRS curves can be well fitted, indicating a direct tunneling transport mechanism^26^. Typically, for metal/insulator/metal junctions, when $\text{V}>\phi$ (where $\phi$ is the barrier height), a change occurs in the barrier shape from rectangular to triangular, leading to a transition of transport mechanism from direct tunneling (DT) to field emission, also known as the Fowler-Nordheim tunneling (FN)^27^. In this high-voltage regime, the current is described by

2 $$\begin{aligned} I\sim V^2 exp\left( \dfrac{4W\sqrt{2m_{e}\phi ^{3}}}{3\hbar qV}\right) \end{aligned}$$

where *W* is the barrier width, $m_{e}$ is the effective electron mass in the oxide, $\hbar$ is the reduced Planck’s constant, $\phi$ is the junction barrier height and *q* is the electronic charge. The transition from DT to FN has been reported in RRAM (resistive random-access memory) devices^28^. A close fit of Eq. (2) is often considered an indication of trap-assisted tunneling (TAT), which has been verified a predominant current transport mechanism in RRAM devices. In Fig. 3c, we plot ln(*I*/$V^{2}$) as a function of 1/*V* at room temperature, which shows clear transitions from DT to FN at $V_{T}\sim 0.83\text{ V}$ (HRS) and 0.64 V (LRS). In the present study, a control sample with fully oxidized $\text{Al}_{2}\text{O}_{3}$ barrier exhibits an MR ratio of 30$\%$ at RT as prepared (see Fig. 3d), and the I–V curve shows no resistive switching. Compared to the I–V curve of the MTJ with partially oxidized barrier (Fig. 1), we conclude that the resistive switching is likely resulted from the oxygen deficiencies created during the preparation process.

**Figure 4:**
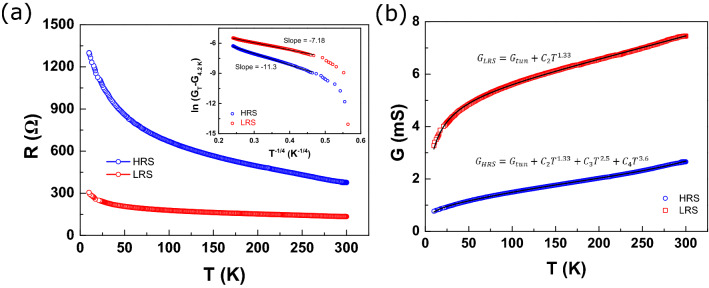
(**a**) The resistance–temperature curves for HRS (blue open circles) and LRS (red open squares). The inset shows the plot of ln($\triangle$G) versus $\text{T}^{-1/4}$ for HRS and LRS with fitting results (solid lines) by using a three-dimensional hopping model. (**b**) The fits of conductivity as a function of temperature for HRS and LRS by a model combining both tunneling and hopping transports.

More details of the transport mechanism can be obtained by analyzing the temperature dependent characteristics. Figure 4a gives the temperature dependence of the MTJ junction resistance. The measurements were performed at a constant current mode at 10 μA, which corresponds to the low voltage regime (from $\sim$ 4.7 to 13 mV for HRS, and from $\sim$ 1.67 to 5 mV for LRS). Both the HRS and the LRS resistances increase with decreasing temperature between 300 K and 10 K, indicating a semiconducting behavior in this temperature range for both resistance states. In the inset of Fig. 4a, we have plotted the temperature dependence of conductance of the MTJ by applying the three-dimensional hopping model:

3 $$\begin{aligned} G(T)\propto exp(AT^{-1/4}) \end{aligned}$$

where *G*, *T*, and *A* denote the conductance, temperature, and a constant related to the bulk-related inverse localization radius ($l_{loc}$), respectively^29^. If the three-dimensional hopping model is obeyed, the relation between ln($\triangle G$) and $T^{-1/4}$ should be linear. Linear fits are obtained between RT and 25 K for both LRS and HRS. This linear behavior is a typical characteristic of inter-particle hopping in metal/semiconductor/metal thin films^30,31^. A close examination of the inset of Fig. 4a shows that the LRS curve agrees to the hopping model better than the HRS. A more complicated analysis combining the inter-particle tunneling and the higher-order inelastic hopping terms is described by Eq. (4), and the fitting results are shown in Fig. 4b for both HRS and LRS.

4 $$\begin{aligned} G(T)=G_{tun}+G_{hop}=G_{0}e^{-(\triangle /T)^{1/2}}+\Sigma C_{n}T^{\gamma } \end{aligned}$$

here $G_{tun}$ is the tunneling conductance, $G_{hop}$ is the spin-independent higher-order inelastic hopping conductance, $G_{0}$ and *C* are free parameters, $\triangle =4E/k_{B}$, where *E* is the tunneling activation energy, $k_{B}$ is the Boltzmann constant, and $\gamma = N-[2/(N + 1)]$ with *N* indicating the number of localized states in the barrier^32^.

The temperature dependence of conductivity at either LRS or HRS can be described by Eq. (4), which implies that the transport mechanism comprises partially the thermal-assisted inelastic hopping through inter-particles. As seen in Fig. 4b, our experimental data are well fitted by Eq. (4) with $\gamma = 1.33$ ($N = 2$) at LRS, suggesting that the conduction is contributed mainly by the tunneling channels and the second-order hopping. As for HRS, the best fit contains not only the second-order hopping ($\gamma = 1.33$) but also the higher-order terms (i.e. third-order with $\gamma = 2.5$, and fourth-order with $\gamma = 3.6$). In metal oxide based devices, the conduction rises primarily through the conduction channels formed in the regions with high concentration of oxygen vacancies ($V_{O}$). Our results show that in addition to the tunneling channels, higher-order inelastic hopping due to the presence of localized states in the $\text{AlO}_{x}$ barrier also contributes significantly to the charge transport process. Such transport process is intriguing in MTJs and nanoparticle networks exhibiting sequential and co-tunneling characteristics, especially at low temperature^33,34^. Further studies at low temperature are on demand to gain insights into the temperature and bias dependences of the sequential and co-tunneling regime.

By correlating the results of Figs. 3 and 4, we conclude that the high MR ratio at HRS corresponds to the insulating regime of the device where MR arises from the tunneling effect, whereas the low MR ratio at LRS is due to the high level of localized/trapping states. In such a case, the magnetoresistance results mainly from the hopping of spin-polarized electrons through the localized states.

## Conclusion

In summary, we have demonstrated that in an $\text{AlO}_{x}$-based MTJ device, both resistive switching and magnetoresistance are observed and are electrically tunable. The room-temperature bipolar resistive switching ratio is $\sim$ 202$\%$ and the MR ratio is 10$\%$ and 4$\%$for HRS and LRS, respectively. The temperature dependence of resistance suggests that the switching mechanism is not the typical filament-type switching. The fitting of conductance to a model combining the inter-particle tunneling and the higher order hopping channels reveals that by electrically altering the oxygen vacancies in the $\text{AlO}_{x}$ barrier, the magnetoresistance can be tuned simultaneously. The results lead to a promising possibility of integrating MTJ into the future multi-bit spintronic memory devices.
